# Design and Manufacturing of a Metal-Based Mechanical Metamaterial with Tunable Damping Properties

**DOI:** 10.3390/ma15165644

**Published:** 2022-08-17

**Authors:** Konstantin Kappe, Jan P. Wahl, Florian Gutmann, Silviya M. Boyadzhieva, Klaus Hoschke, Sarah C. L. Fischer

**Affiliations:** 1 Fraunhofer Institute for High-Speed Dynamics (EMI), Ernst-Zermelo-Str. 4, 79104 Freiburg, Germany; 2 Fraunhofer Institute for Nondestructive Testing (IZFP), Campus E3 1, 66123 Saarbrücken, Germany

**Keywords:** energy dissipation, energy absorption, bistability, metamaterials, additive manufacturing, damping

## Abstract

In the present work, a novel concept for metallic metamaterials is presented, motivated by the creation of next-generation reversible damping systems that can be exposed to various environmental conditions. For this purpose, a unit cell is designed that consists of a parallel arrangement of a spring and snap-fit mechanism. The combination of the two concepts enables damping properties one order of magnitude higher than those of the constituting metal material. The spring element stores elastic energy while the snap-fit allows to absorb and dissipate energy and to reach a second stable state. Different configurations of single unit cells and connected cell assemblies are manufactured by laser powder bed fusion using Ti6Al4V powder. The dimensioning is supported by finite element modelling and the characteristic properties of the unit cells are studied in cyclic compression experiments. The metamaterial exhibits damping properties in the range of polymeric foams while retaining its higher environmental resistance. By variation of selected geometrical parameters, either bistable or self-recovering characteristics are achieved. Therefore, a metamaterial as an assembly of the described unit cells could offer a high potential as a structural element in future damping or energy storage systems operating at elevated temperatures and extreme environmental conditions.

## 1. Introduction

Dissipation and absorption of mechanical energy play an important role in many situations. They are essential for our safety in everyday life, for example, in crash bumpers [1], bicycle helmets [2,3] or in the presence of operating machines and engines where critical vibrations such as resonance oscillations are ubiquitous [4]. Therefore, technical damping solutions are needed. While there are many approaches, they all come with unique restrictions. Damping systems based on the intrinsic damping properties of materials [5], for example, using viscoelastic materials or plastic deformation of metals, do not allow the design for an application requiring at the same time resistance to extreme environmental conditions, dissipation or absorption of a large amount of energy and reversibility. As a result, more complex mechanic and mechatronic systems are developed leveraging, for example, friction-based damping systems [6,7] or hydraulic damping [8,9]. However, these are costly and involve complex electronic infrastructure.

In order to attain properties exceeding those of the natural constituent materials, the design of mechanical metamaterials represents a promising approach for novel damping systems. The underlying idea is the periodic arrangement of geometric unit cells to form a material with properties beyond the bulk properties [10,11,12,13,14,15]. They can achieve, for example, a negative Poisson’s ratio [16], negative stiffness [17], remarkable elastic deformation [18] or twist under pressure loading [19]. Due to the rapid development of additive manufacturing technologies, the possibilities and research to develop novel metamaterials with complex and filigree structures have increased substantially in the recent past [20,21,22]. Multistable mechanical metamaterials are promising to realize recoverable energy absorbing behavior [17,23,24,25,26,27,28,29,30,31,32]. Thereby, the movement from a mechanism’s initial state to another stable state through external forces enables the reversible storage of strain energy by elastic deformation. Multistable mechanisms can generally be classified into latch-lock mechanisms [33], hinged multi-segment mechanisms [34], and buckling mechanisms exploiting residual stress [35]. For mechanical metamaterials, approaches primarily concentrate on utilizing stability phenomena such as buckling of beams or plates [24], curved beams [27,28], and even more complex three-dimensional arrangements of beams in unit cells [25,29].

However, in the above-mentioned studies, the design of the multistable materials is restricted to polymeric materials. The difficulty designing similar mechanisms with metals is related to the typically lower elastic strains that are possible. This limits the flexibility of monolithic structures and therefore requires the development of novel concepts that specifically consider the mechanical properties of metals and restrictions of metal additive manufacturing technology. An interesting approach consisting of metallic honeycomb unit cells with integrated friction elements has been published by Garland [36]. Under load, reversible energy dissipation was achieved through friction among the contact areas and deflection of the structural components, achieving a damping factor *η* = 0.507, which is higher than inherently possible with the bulk metal. However, the storage of energy and, therefore, the realization of two stable states has not been a part of the work.

The novelty of the present work consists of the combination of the two afore-mentioned concepts, bistable behavior and friction-based elements, integrated into a metallic metamaterial to reach damping properties 1000 times higher than the constituting metal. A new unit cell is designed combining a spring and a snap-fit element. The spring element stores elastic energy and ensures the monolithic connection of the joining partners. The snap-fit allows the mechanism to absorb as well as dissipate energy and to reach a second stable state. Unit cells with different parametrization were designed supported by finite element modelling, to show the transition between self-recovering and bistable behavior. The samples were additively manufactured to study the energy storage and damping properties in cyclic compression tests experimentally. In addition, the authors extended the experiments to unit cells in series and in parallel configuration to show the scalability of the concept. Thus, the study contributes to the development of novel mechanical metamaterials with exceptional properties using metal as base material. This requires new design concepts and ideas, especially due to the typically low elastic strains that are possible.

## 2. Materials and Methods

### 2.1. Design Restrictions of a Metal Unit Cell

When dimensioning the unit cell, restrictions of additive manufacturing as well as the mechanical material properties have to be considered. The geometries were designed for processing the titanium alloy Ti6Al4V in the laser powder bed fusion (LPBF) process. A Young’s modulus of about 110 GPa and a yield strength of 1100 MPa are specified for the material so that a permissible elastic elongation of 1% is assumed. CAD-models were created using CATIA V5-6R2018 (Dassault Systèmes SE, Vélizy-Villacoublay, France) with dimensions of a single unit cell of 10 × 10 × 11.65 mm^3^. To ensure manufacturability and avoid support structures needed for LPBF inside the unit cell, self-supporting segments are added to the structure at critical overhanging surfaces. Furthermore, cutouts were designed to avoid thermal distortion. Constructive gaps of 100 µm between the structural parts were taken into account to prevent unwanted merging.

### 2.2. Additive Manufacturing

All specimens were produced on an EOS M 100 (EOS GmbH, Kralling, Germany) LPBF machine with a 200 W laser unit (YLR-series, CW-laser, wavelength 1070 nm) in 20 µm layers. To realize the constructive gap of 100 µm, the process parameters were tuned and set to a scanning speed of 2000 mm s^−1^ and a laser power of 50 W. For the production, recycled Ti6Al4V powder (EOS Titanium Ti6Al4V) sieved with a 63 µm mesh was used. The unit cells were printed in a 45° orientation to the build plate to reduce acute angles. An argon-based inert gas atmosphere of O_2_ < 0.1% was applied in order to avoid oxidation. The test specimens were examined in the as-built state without further surface or heat treatment.

### 2.3. Finite Element Modeling

Numerical simulations were carried out to analyze occurring principal stresses as well as structural behavior of the snap-fit and spring mechanism during compression. The effect of large deformations on the structural-mechanical behavior was computed in non-linear, implicit finite element (FE) calculations based on the Optistruct (Version 2019.2, Altair Engineering Inc., Troy, MI, USA) finite element code. Solid hexahedral elements with a maximum edge length of 0.1 mm were used. A linear elastic material model based on mechanical properties of Ti6Al4V with Young’s modulus of 110 GPa, Poisson’s ratio of 0.3, and density of 4.4 g cm^−3^ was chosen. The contact surfaces were modeled with a surface-to-surface contact considering continuous sliding. Using symmetries, only one side of two symmetrically identical arranged snap-fits was simulated. A rigid body element was used to impose a uniform displacement on the bounding surface that controls the cycle of compression and extension of the cell in a quasi-static load case. The loading and unloading of the spring were calculated by specifying the displacement of the snapping process in the experiments, i.e., 1.75 mm. Further information about the simulation of the unit cell mechanisms is available in Appendix A.

### 2.4. Mechanical Testing

Quasi-static compression and cyclic compression tests were performed on a AllroundLine Z250 (ZwickRoell AG, Ulm, Germany) testing machine with testControl II measurement and control electronics equipped with a 1 kN load cell. The quasi-static test procedures were set up by means of the testXpert III testing software, defining five consecutive displacement-controlled load cycles at a constant testing speed of 1 mm min^−1^. The loading and unloading tests were limited by reaching a limit force immediately after the snap-fits are pressed against the base of the cell in load direction or against the joining partner’s barrier in the direction of unloading. To analyze the possible change in mechanical behavior due to an increased number of loading cycles, selected samples were subsequently loaded up to 100 times at an increased test speed of 10 mm min^−1^ in additional measurements. The occurring forces and the displacements were measured at a frequency of 10 Hz.

### 2.5. Nondestructive Characterization

Computed tomography (CT) measurements were performed with a custom-built CT scanner at Fraunhofer IZFP (Saarbrücken, Germany) equipped with an X-ray tube of type X-ray Works XWT-240 SE and a PerkinElmer 1610 detector with operating parameters 230 kV and 40 µA, a voxel edge length of 10 µm, exposure time per projection of 2 s and 1600 projections in total. Reconstruction was performed with two software tools, a custom tool VolumePlayerPlus 8.1.5 developed by Fraunhofer IZFP (Saarbrücken, Germany) and VGStudio MAX 3.4 from Volume Graphics (Heidelberg, Germany). CT measurements were performed on one sample of type V2.2 in two different states, as manufactured and after approximately 100 cycles of loading.

## 3. Results

### 3.1. Design of Metamaterial for Recoverable Energy Absorption and Dissipation

**Figure 1:**
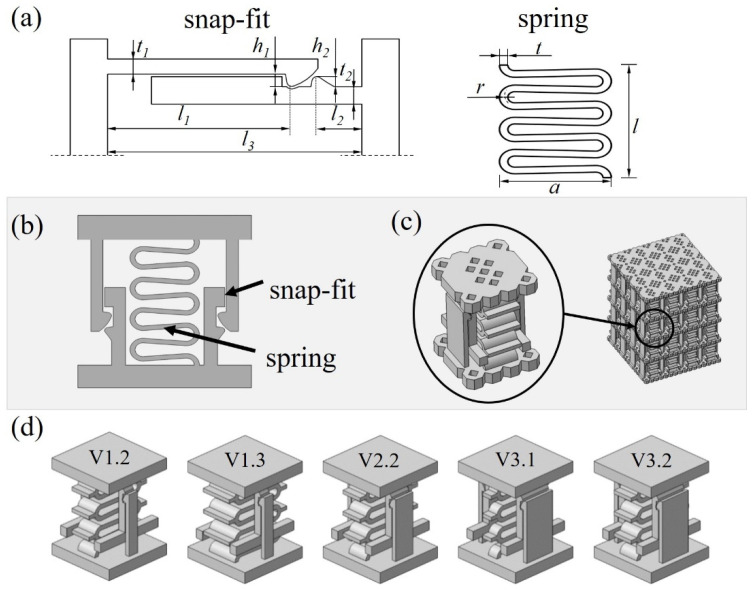
(**a**) Main mechanisms of the unit cell and design parameters, parameter set described below in the main text, (**b**) 2-D concept of the reversible energy absorbing metallic unit cell consisting of the spring and snap-fit mechanism, (**c**) fabrication model of the unit cell and metamaterial assembly, (**d**) cell configurations with varied spring and snap-fit width as variable factors. The designation VA.B corresponds to: A—the snap-fit width and B—the spring width with 1.5 mm (1), 3 mm (2), and 4.5 mm (3).

The novel mechanical metamaterial designed, optimized, and validated in this study consists of the parallel arrangement of two mechanisms shown in Figure 1a, a snap-fit and a spring. These mechanisms have two main objectives, to dissipate energy by friction and to enable the programming of bistability of the unit cells depending on the requirements of a certain application. The concept of the 2-D and 3-D unit cell is shown in Figure 1b,c. Parametrization of the snap-fit and spring allows a transition between bistable or self-recovering behavior that will be further studied numerically and experimentally in this work. In a bistable behavior, the required force of the snap-fit mechanism is greater than the restoring force of the spring and the unit cell remains in a second stable position. For the self-recovering behavior, the force of the spring exceeds the restraining force of the snap-fit mechanism so that the system returns to its initial state without external force.

In order to ensure repeatability in the compression tests, the structures are designed for purely elastic deformation, avoiding exceedance of the material’s yield strength of 1100 MPa during the compression and retraction motion of the unit cell. Based on the 2D-models as shown in Figure 1a, a numerical simulation was performed to predict stresses within the geometry up to the full compression at 1.75 mm displacement. The width of the snap-fit and the spring were not considered, as the unit cell was designed as an extrusion of the 2D-models and changing the widths will only affect the absolute force values but not the stresses in the structure for the prescribed displacement. To ensure that the maximum stresses in the structure do not exceed 80% of the yield strength, the following geometric parameters were chosen for all structures presented in this work:

- Parameters of the spring element: 7 windings, *t* = 0.6 mm, *l* = 10.15 mm, *a* = 8 mm, *r* = 0.8 mm;
- Parameters of the snap-fit mechanism: *t*_1_ = 0.6 mm, *t*_1_ = 0.8 mm, *h*_1_ = 0.4 mm, *h*_2_ = 0.4 mm, *l*_1_ = 7.35 mm, *l*_2_ = 1.8 mm and *l*_3_ = 10.15 mm.

Detailed information about the mechanical design of the unit cells is available in Appendix A.

To program the macroscopic properties of the metamaterial and investigate the damping as well as stability properties of the unit cell, two input factors, namely the snap-fit width (A) and width of the spring (B), are varied in three levels, with dimensions of 1.5 mm (1), 3 mm (2), and 4.5 mm (3). From the possible combinations, 5 different specimen geometries coded by VA.B shown in Figure 1d were selected to determine the influence of the parameters. Other parameters, in particular of the joining geometry, are also suitable for modifying the mechanical behavior of the unit cell and thus of the metamaterial, but require further investigation and are not addressed within this study.

### 3.2. Analytical Model

In a first step, the behavior of the unit cell is described analytically with a superposition of the external forces required for displacing the snap-fit and the spring:

(1) $$F_{cell}=F_{spring}+F_{snap-fit}$$

**Figure 2:**
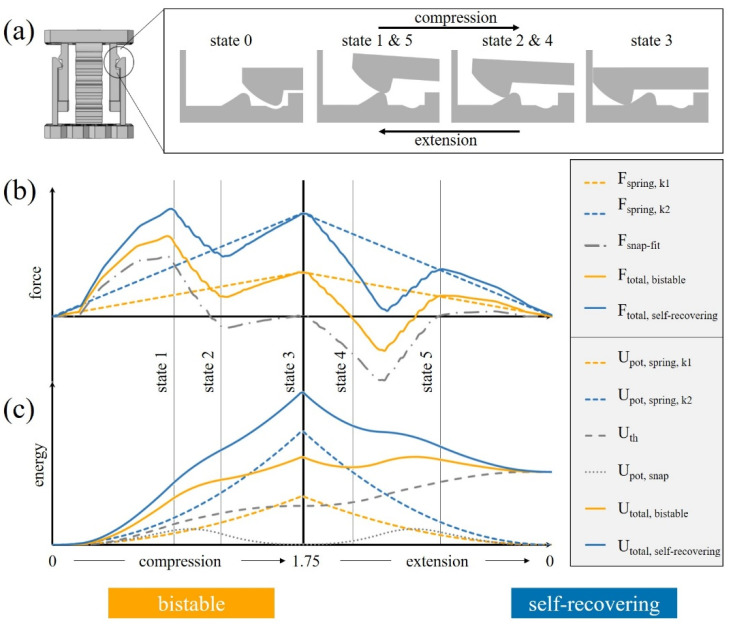
(**a**) Illustration of the deformation of the snap-fit between the fully relaxed position (state 0) and fully compressed position (state 3). Analytical model of the friction-based metamaterial for two exemplary unit cells with identical snap-fit properties and different spring width and spring constants *k*_1_ < *k*_2_, resulting in a bistable (orange, spring *k*_1_) and self-recovering (blue, spring *k*_2_) unit cell. (**b**) Force–displacement curves of an exemplary snap-fit mechanism (*F_snap-fit_*, dashed/dotted line), springs with different spring constants *k*_1_ < *k*_2_ (*F_spring_*, dotted line), and the force superposition for the two unit cells (*F_total_*, solid line). (**c**) Energy–displacement curves representing the potential and thermal energies (*U_pot,spring_*, *U_pot,snap_*, and *U_th_*, dotted and dashed lines) and the resulting total absorbed energy (*U_total_*, solid line).

The spring force increases and decreases linearly between the initial state (state 0, Figure 2a) and the fully compressed state (state 3), as expected according to Hooke’s law. The slope of the curve and the maximum force reached at full compression are determined by the dimensions (in particular by the width of the spring) and results in different spring constants.

The force contributed by the snap-fit mechanism depends on the position of the snap-fit and the direction of loading and is composed of the frictional force and the contact force against the direction of motion. Starting from the initial state (state 0), the force contributed by the snap-fit increases until the snap-fit reaches the top of the hook geometry (state 1). Further displacement results in a decrease of the force caused by the elastic strain energy being released in the deflected snap-fit arms and the decreased contact force to reach a minimum (state 2) before full compression (state 3) where the force levels out. Depending on the dimensions of the hook geometry (Figure 1a) and the coefficient of friction, the force can become negative, i.e., tensile forces occur in the snap-fit. When releasing the cell, the snap-fit force *F_snap-fit_* decreases, as it is under tension due to contact and frictional forces (state 4), before being superposed with an increase in force due to deflection of the snap-fit, as it surpasses the hook geometry (state 5). After this state, frictional forces and stored elastic energy are in equilibrium until reaching the fully relaxed initial position (state 0). Due to a non-symmetrical snap-fit geometry, the force required to return to the initial state differs from the force during the compression progress.

The effective force that the unit cell is subject to results from the superposition of the two aforementioned phenomena (Equation (1)). The spring width and snap-fit width influence the magnitude of the two contributions and, thus, the transition between self-recovering and bistable behavior of the unit cell. As exemplarily shown in Figure 2b for two unit cells with the same snap-fit geometry and coefficient of friction but different spring width, respectively spring constants (*k*_1_ *< k*_2_), the spring’s elastic restoring force (*F_spring_*) is smaller than the force required for the snap-fit mechanism to exceed the hook, and the overall force (*F_total_*) at state 4 is negative in the case of bistable behavior while remaining positive in case of self-recovering cells.

Furthermore, the characteristic behavior of the presented unit cell can be investigated from an energetic point of view:

(2) $$U_{total}=\underset{U_{pot}}{\underbrace{U_{pot,spring}+U_{pot,snap}}}+U_{th}$$

*U_pot_* is the energy resulting from the potential energy of the spring *U_pot,spring_* and the snap-fit mechanism *U_pot,snap_*. The potential energy of the spring is stored reversibly by its elastic deformation. The potential energy of the snap-fit is stored in the form of strain energy due to the elastic deformation of the snap-fit structure, which, after the hook surpasses the hump and via snapping into the cavity, is released again. This results in two local minima in the fully relaxed position (state 0) and fully compressed position (state 5) (Figure 2c). *U_th_* represents the thermal energy caused by the frictional contact and increases steadily during the motion, regardless of the direction of loading. Furthermore, the deflection of the snap-fit arm influences the normal force and, thereby, the frictional energy. Detailed information about the energy characteristics of the unit cells is available in the supporting information in Appendix B.

The differing behavior of a self-recovering or a bistable cell can be described based on the change in potential energy during a compression cycle. Bistability is reached at the point where the potential energy has a local minimum to be overcome by an external force:

(3) $$\frac{\partial U_{pot}}{\partial d}=0,\;\frac{\partial^{2}U_{pot}}{\partial d^{2}}>0$$

As exemplarily shown in Figure 2c, the effective energy of a bistable cell *U_total,bistable_* reaches a local minimum (state 4) followed by a local energetic maximum that requires external energy to be overcome during the extension process, while *U_total,self-recovering_* is characterized by a steady decrease and does not require external energy to reach the uncompressed state.

### 3.3. Numerical Simulations

A numerical study of a full compression cycle was performed for the five unit cell configurations (Figure 1d) as well as the respective snap-fit and spring elements, to predict whether a bistable or self-recovering behavior can be expected. Since the coefficient of friction is unknown, simulations were performed for two coefficients *µ* = 0.4 and *µ* = 0.7, to compute a range of the expected force values. 

An overview of the results is shown in Table 1. The maximum force of the snap-fit mechanism (*F_snap,e_*) and the associated spring force (*F_spring,e_*) are added together to define the force of the cell (*F_cell,e_*) during extension. As described by the analytical model in Section 3.2, *F_cell,e_* refers to the force that needs to be applied to return the system to its initial state where a positive sign and a negative sign indicate bistable and self-recovering behavior, respectively.

The simulations predict that the values of the restoring force *F_cell,e_* increase with increasing ratio *b_snap_/b_spring_*. Based on the results, self-recovering behavior is expected for V1.3 and bistability for V2.2, V3.1, and V3.2. For cell V1.2, it is ambiguous whether the behavior is bistable or self-recovering, as the sign of *F_cell,e_* spans from −8 to 1 N for the range of frictional coefficients. Generally, it can be deduced that an increase of the ratio *b_snap_/b_spring_* leads to a higher restraining force, which prevents the return into the initial state and therefore increases the chances for the unit cell to display bistable behavior. Based on the numerical simulations, *b_snap/_b_spring_* is suitable as a design criterion for the transition between both behaviors with a threshold expected for *b_snap_/b_spring_* of around 0.5. This threshold is influenced by many parameters including the friction coefficient and, therefore, further simulations are necessary when transferring the proposed concept, for example, to a different parametrization, manufacturing strategy or material.

### 3.4. Cyclic Mechanical Testing

To validate the numerical simulations and the analytical model presented in the previous two sections, five parametrized unit cells as shown in Figure 1d were additively manufactured and experimentally investigated in cyclic compression tests. The experiments were performed with three samples of each unit cell geometry.

**Figure 3:**
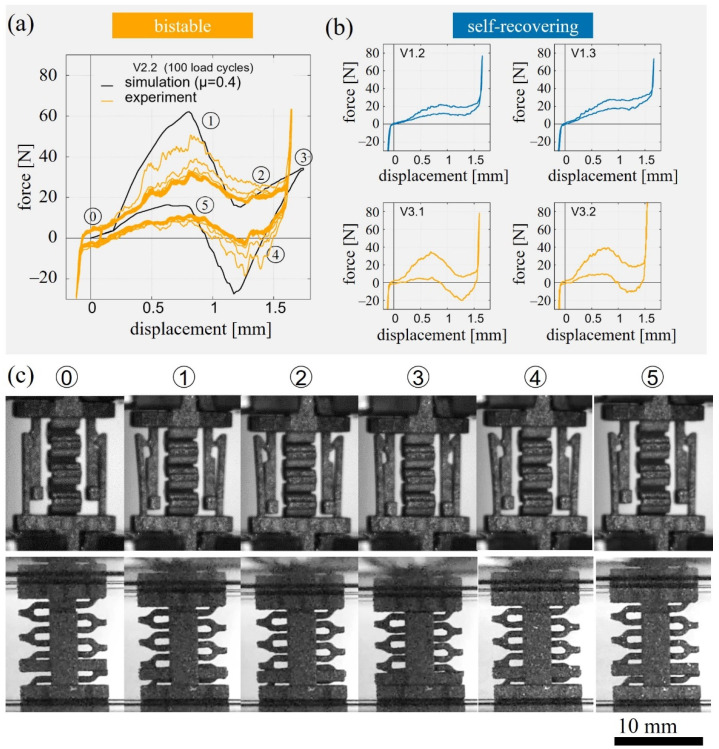
Force–displacement diagrams of frictional snap-fit cells V1.2, V1.3, V2.2, V3.1, V3.2 during cyclic compression and representative optical micrographs between the fully relaxed position (state 0) and fully compressed position (state 3); (**a**) experimentally obtained force–displacement curves during 100 load cycles for sample V2.2 (orange curve) and corresponding simulation results (black curve); (**b**) force–displacement curve of the 4th load cycle for the remaining geometries: V1.2, V1.3, V3.1, V3.2; (**c**) side views of the unit cell V2.2 in characteristic deformation states during cyclic compression.

Generally, the cyclic compression tests of all unit cells have a characteristic shape associated with the different deformation states of the unit cell (Figure 3a,c). Starting from the fully relaxed state ⓪, the force increases until *F*_1_ at state ①, where the maximum deflection of the snap-fit arms is almost reached. Subsequently, the force decreases as the hook geometry is overcome to reach a local minimum *F*_2_ in state ②, then further increases with steady compression of the spring until the fully compressed state ③ with force *F*_3_. A rapid increase in force is measured when the snap-fits are pressed against the base plate. This occurred even before reaching the maximum displacement of 1.75 mm for an ideal cell. During unloading to the extended initial state ⓪, a local extremum with the force *F*_4_ is reached in state ④ when the hook geometry is passed once again. Furthermore, an increase of the force until the local maximum at *F*_5_ is observed when passing state ⑤.

In all experiments, the magnitude of the forces decreased with increasing cycle number and reached a stable state starting with the 4th cycle, as shown exemplarily for cell V2.2 in Figure 3a. Therefore, the force–displacement curves for the remaining geometries are shown for the 4th load cycle only for better comparability (Figure 3b). The corresponding characteristic values of the forces *F*_1_ to *F*_5_ are evaluated based on the 4th load cycle. 

Experiments showed that in agreement with the simulations, the configurations V2.2, V3.2, and V3.3 can be classified as bistable and V1.3 as self-recovering. While the simulations were ambiguous for V1.2, the experiment revealed self-recovering behavior for this unit cell. In the following, the characteristic forces of the different unit cells will be analyzed (Table 2) and compared to the trends expected from the analytical model. 

*F*_1_ increased from V1.2 with 22 N to V3.2. with 39 N, due to the increase in spring and snap-fit width. *F*_2_ and *F*_3_ were highest for V1.3 with 27 N and 38 N, almost equal for V1.2, V2.2, and V3.2 with 18 N and 28 N and the lowest for V3.1 with 7 N and 16 N, respectively. These two forces are mainly determined by the spring width and the experimental results were therefore in good agreement with the analytical model. *F*_4_ was positive for V1.2 and V1.3 which corresponds to self-recovering behavior and negative for V2.2, V3.2, and V3.3, indicating bistable behavior. While there was a pronounced drop of the force at state 4 for V3.2 and V3.3 with −20 N and −11 N, *F*_4_ is only −1 N for V2.2. This indicates that this cell parametrization is close to the design parameters at the transition between both regimes and only a slight decrease of *b_snap_/b_spring_* below 1 could yield self-recovering behavior. *F*_5_ is expected to increase with the spring width and decrease for wider snap-fit mechanism due to higher friction forces. The experiments revealed an increase of *F*_5_ with decreasing *b_snap/_b_spring_* ratio from 6 N for V3.1 to 18 N for V1.3.

Comparing the results from simulations based on a frictional coefficient of *µ* = 0.4 and the experiments for the unit cell V2.2 (Figure 3a), it can be observed that the simulations overpredicted the forces even for the initial loading cycle with 30.8 N as measured in experiments compared to 60.9 N from simulations. It is worth mentioning that the overprediction of the forces in the simulation can be observed for all specimen geometries.

Besides the decrease of the force over the first three cycles, an examination of the snap-fit surfaces revealed a glossier appearance of the surface after cyclic loading. All phenomena indicate a strong influence of the coefficient of friction and the surface topography on the function of the snap-fit mechanism of the unit cell. 

### 3.5. Friction Behavior

**Figure 4:**
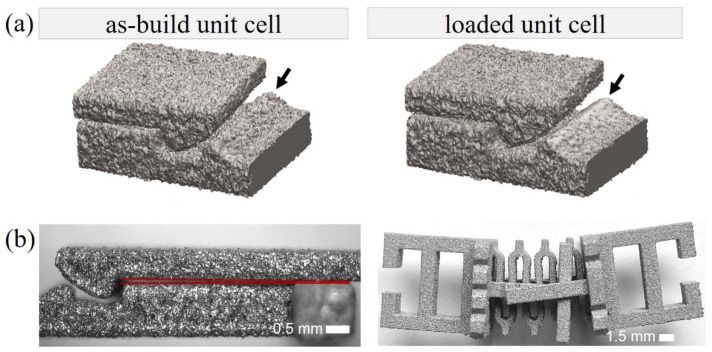
CT-reconstructions and optical micrographs of different sample variations induced by manufacturing or experiments. (**a**) Reconstruction of CT-scans of a V2.2 snap-fit as-built (**left**) and a loaded (**right**). (**b**) Representative optical micrographs showing distortion of the manufactured unit cell.

In order to further explore the factors influencing the coefficient of friction of the snap-fit, nondestructive X-ray tomography scans were performed on a unit cell V2.2 as manufactured and after approximately 100 loading cycles, to study the topographical changes of the snap-fit surface due to the frictional abrasion occurring during loading. The reconstructions of the snap-fit (Figure 4a) revealed that the as-built structure exhibits a higher surface roughness compared to the loaded unit cell where less attached powder particles and a smoother contact area were observed. Furthermore, optical micrographs illustrate that, although the samples were optimized for additive manufacturing, deviations from the ideal geometry due to the release of manufacturing-induced residual stresses occur. Distortions of the filigree snap-fit arms were observed (Figure 4b), which reduce the effective hook geometry and, therefore, the normal force. Similarly, a twist of the whole unit cell could be seen, probably likewise due to process-induced distortions (Figure 4b).

### 3.6. Damping Behavior

In the following section, the damping potential of the proposed metamaterial is evaluated. The damping coefficient and effective elastic modulus of each unit cell were determined based on findings from the mechanical experiments. Since the unit cell exhibits a nonlinear elastic structural behavior, the elastic modulus *E_unit,cell_* is calculated using the secant method with the measured compression force *F*_3_ and the associated macroscopic change in length *d*_3_ up to state 3 (cf. Figure 3a):

(4) $$E_{unit,cell}=\frac{F_{3}}{d_{3}}$$

The structural loss coefficient η is calculated as a function of the ratio of the absorbed energy U and the dissipated energy ∆*U* [5]:

(5) $$\eta =\frac{∆U}{2\pi U}$$

The dissipated energy ∆*U* corresponds to the area between the loading and unloading curves. The evaluation of the force curves was carried out for the elastic compression of the cells up to *F*_3_, to avoid a rapid increase in the force caused by the snap-fits coming in contact with the base plate. The absorbed energy *U* results from the superposition of the potential energy of the spring and the snap-fit and the thermal energy until contact with the base plate occurs and is calculated by integration of the force–displacement curve of the experimental results. The results are summarized in Table 3.

The absorbed energy increased with increasing width of the snap-fit and the spring from 25 mJ for V1.2 up to the maximum of 36 mJ for V3.2. As both V1.2 and V3.2 exhibit a snap-fit width of 3 mm, it can be deduced that the elastic spring energy has a major influence on the absorbed energy. The dissipated energy, on the other hand, is governed by the snap-fit characteristics, and the width of the spring had no significant influence due to the linear loading and unloading curve (cf. Figure 2). The dissipated energy of the unit cells increased with increasing width of the snap-fits from 9 mJ for V1.2 to 33 mJ for V3.2 due to the increased normal force resulting from the deflection of the frictional snap-fit elements.

The loss coefficient was lowest for the self-recovering unit cells at about 0.05–0.06 and increased by a factor of two to four for bistable cells. η was highest for V3.1 with 0.191 and even exceeded the value of $\eta_{∆U=U}=1/2\pi$, indicating that more energy was dissipated in one cycle than was absorbed in total during loading. This can be attributed to the novel friction-based metamaterial design with the bistable snap-fit mechanism requiring an external force in the opposite direction of loading in order to go back to the initial state.

The elastic modulus of the unit cells varied from 1.23 MPa for V3.1 to 2.69 MPa for V1.3, which corresponds to an approximate 100-fold decrease compared to the Young’s modulus of the bulk material with 110 GPa. Since the modulus is calculated using the secant method based on the compression force *F*_3_ and the corresponding displacement *d*_3_, it is equivalent to the spring stiffness.

### 3.7. Arrangement of Unit Cells in Parallel and in Series

**Figure 5:**
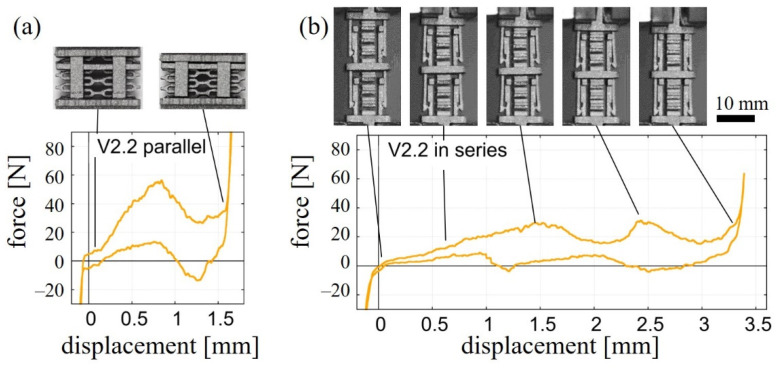
Force–displacement diagrams of arrangements of two V2.2 cells during cyclic compression. (**a**) Representative curve for a parallel arrangement. (**b**) Representative curve for an arrangement in series.

In addition to single unit cells, cell arrays with two unit cells of type V2.2 were manufactured to analyze the effect of the arrangement of unit cells in series and parallel and to demonstrate scalability of the concept. For the configuration in parallel, the experimental results in Figure 5a confirmed that the forces add up according to the number of parallel cells while showing peaks at similar deformation states as in the case of a single unit cell (Figure 3a). The first maximum force *F*_1_ was determined to be 30.8 ± 1.2 N and 58.1 ± 1.7 N for the single cell and parallel cell, respectively. Additionally, the snap process of the two parallel cells occurs simultaneously.

For unit cells arranged in series, as shown in Figure 5b, the shape of the force–displacement curve changed in comparison to a single unit cell. A sequential snapping was observed for the bistable unit cell V2.2 and, thus, multiple stable states can be achieved. Here, the macroscopic displacement between successive snapping processes is greater with fewer unit cells in their bistable state, as the applied strain is distributed across the cells before snapping. The force level of the two local maxima of the serial arrangement with 29.3 ± 0.5 N for the first and 28.2 ± 2.2 N for the second are similar compared to *F*_1_ = 30.8 ± 1.2 N of the single unit cell. Since the characteristic of the single unit cell does not change, the damping properties as well as the elastic modulus are comparable to the single cell.

## 4. Discussion

The analytical, numerical, and experimental results presented show that the concept of the unit cell is suitable for the goal of energy dissipation and absorption. The damping properties and characteristics of the unit cell can specifically be programmed into the materials by varying the geometric parameters, namely the width of the spring and snap-fits. In the future, further geometric parameters, constituent material properties, and frictional effects can be studied to extend the parameter space. 

Based on the five cell configurations studied in detail, bistable and self-recovering behavior could be predicted by simulations and validated by experimental results. Cell geometry V2.2 represents an interesting case as it illustrates the experimental design threshold between self-recovering and bistable behavior due to the changing coefficient of friction throughout the first four loading cycles.

The force–displacement curves from experiments exhibited similar shapes compared to simulations (cf. Figure 3a). The steady force–displacement curves after the fourth load cycles indicate that the components of the unit cells do not deform plastically during the tested number of cycles. However, the experimental results overall show an unexpected low force level (cf. Table 2) and the peaks at the state of maximum deflection of the snap-fits are significantly attenuated (cf. Figure 3). This suggests that the effective friction coefficients and the size of the tested cells differ from the actual CAD models.

Several factors contribute to these discrepancies and need to be taken into account for further studies of metallic metamaterials:

1. Manufacturing tolerances play an important role in the filigree structures as these result in comparatively large relative deviations in the effective shape.
2. Surface roughness is another artefact of the additive manufacturing process in a metal powder bed as it results in a rough surface layer, as mentioned by Garland [36] and Qui [37]. Microscopic optical images and CT-scans of the unit cells confirmed particle attachment on the sample surfaces. Poorly melted particles were removed during the first loading cycles due to the frictional contact, which reduces the coefficient of friction through wear and rounds off the joining surfaces, reducing the effective hook depth of the snap-fits (see Figure 4a).
3. Distortion of cells occurs due to the release of thermal residual stresses caused by the manufacturing process. For the snap-fit, deviations result in a reduction of the overhanging distance of the hook and thereby influence the normal force of the frictional contact.
4. Warpage is caused because the compliant spring allows the samples to bend around the axis of the extrusion direction after removal of the supporting structures needed for the manufacturing process. This leads to a prestress in the samples during the experimental setup when clamping them in a position with parallel base plates.

A prediction of the actual coefficient of friction of the unit cells based on the comparison of present experiments and simulations is not possible. Further investigations of the frictional characteristics of additively manufactured materials and integration of more complex frictional boundary conditions in the simulations are necessary to understand the dynamic phenomena and complex interplay between all factors.

The combination of the single bistable V2.2 unit cell in series or parallel connections shows the scalability of the concept to a metamaterial cell assembly (Figure 5). Parallel arrangement of the cells resulted in a behavior that was in good agreement with the experiments of single cells. The forces scaled by a factor of about two and simultaneous transition of all snap-fit mechanisms through all states was experienced. Connecting cells in series resulted in a multistable behavior since the cells transition through the states sequentially. This results from an uneven distribution of strain in the system due to increased thermal distortion effects. A mirrored arrangement of the spring elements could counteract the thermal warping effect for further manufacturing of cell assemblies. Generally, similar results are expected for the other unit cell configurations, both for the ones with self-recovering as well as with bistable properties.

**Figure 6:**
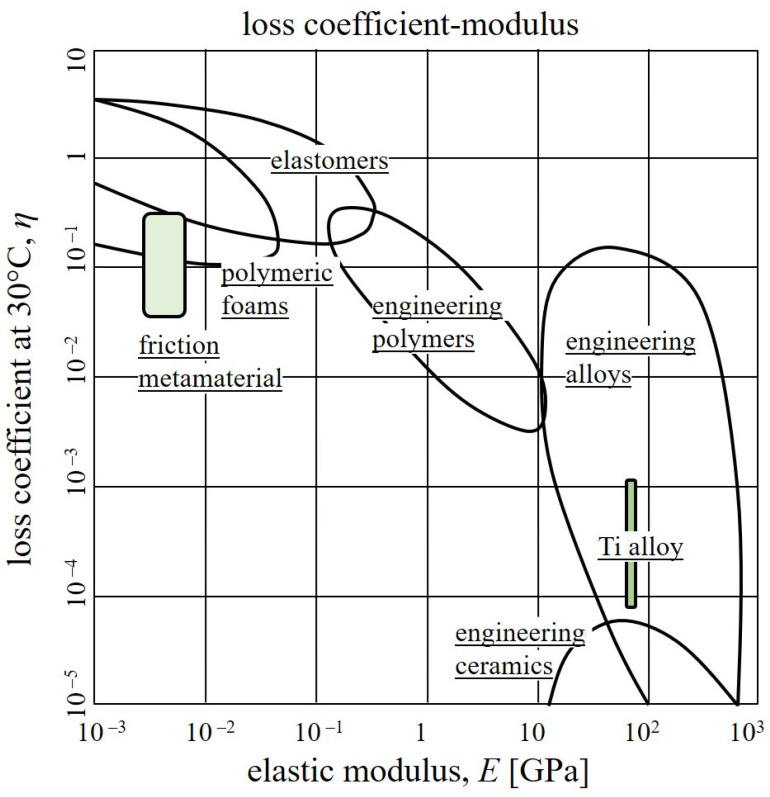
Loss coefficient–elastic modulus diagram of different materials according to Ashby [5] and the developed metamaterial.

Overall, the novel unit cells successfully tailored the mechanical properties and damping properties to become vastly different from the material’s bulk properties. The elastic modulus is significantly reduced compared to the titanium alloy base material with 110 GPa, reaching 1.2–2.7 MPa for the different cell configurations, which is comparable to the stiffness of highly elastic rubber or polymeric foams [5]. The loss coefficient η was increased by more than one order of magnitude from about 10^−4^–10^−3^ for the constituent material to 0.05–0.2 for the metallic metamaterial. The damping properties are thus again increased to the level of polymeric foams, high-damping metal alloys, and engineering polymers [5]. At the same time, the unit cells retain the high temperature resistance of titanium alloys, which is about 1100 °C and about ten times higher than that of conventional elastomers with a working range of up to about 150 °C. The characteristic properties, the elastic modulus, and loss coefficient of the presented metamaterial in comparison to the conventional materials are illustrated in Figure 6, to showcase the unique property combinations achieved with the presented concept. The above-mentioned ranges of material properties can be adjusted by variation of geometrical parameters and thereby can be programmed for numerous applications. In the future, further investigation of the cell design and friction characteristics can yield novel damping solutions for industrial applications.

## 5. Summary

In the present study, a new metallic metamaterial allowing dissipation and absorption of energy through a combination of elastic deformation and friction-based elements is presented. The metamaterial containing a snap-fit mechanism and a spring element was designed and evaluated considering the restrictions of additive manufacturing and the limited elastic deformation of metallic materials. Selected unit cells of the metamaterial were manufactured by laser powder bed fusion of Ti6Al4V and were investigated with both simulation and experiment. A bistable or a self-recovering snap-fit behavior with fully elastic deformation under compression was tailored by tuning geometrical parameters of the cell. The damping properties and compliance of the metallic systems were determined using cyclic compression tests and were found to be similar to those of elastic foams while also bringing the inherent properties of metallic materials such as resistance to environmental influences like heat. Further adjustments of the design parameters will allow the extension of the achievable range of properties.

In conclusion, the design of the proposed metamaterial allows programming its mechanical properties for applications in various use cases such as vibration damping, reusable energy absorber or shape changing systems, even in harsh environmental conditions. This concept opens up new opportunities to design innovative material systems for complex requirements. However, additive manufacturing of the metallic metamaterial is still challenging as the fabrication constraints and tolerances highly influence the functionality of the metamaterials. Development of new adapted process parameters for these filigree structures as well as robust design strategies, which take manufacturing limitations into account, are necessary. In addition, tailored destructive and nondestructive characterization methods need to be investigated to gain understanding about the fatigue behavior and frictional properties of complex metamaterial systems to enable their scalability and reliability for technical applications.

## Figures and Tables

**Table 1:** Mechanical properties of the unit cell designs based on numerical simulations for a friction coefficient *µ* = 0.4 and *µ* = 0.7, where the smaller force value of the depicted range of *Fsnap,e* and *Fcell,e* corresponds to the smaller friction coefficient and vice versa.

Parameter	V1.2	V1.3	V2.2	V3.1	V3.2
*b_snap_*	1.5 mm	1.5 mm	3 mm	4.5 mm	4.5 mm
*F_snap,e_*	16 to 25 N	16 to 25 N	31 to 50 N	47 to 74 N	47 to 74 N
*b_spring_*	3 mm	4.5 mm	3 mm	1.5 mm	3 mm
*F_spring,e_*	24 N	35 N	24 N	16 N	24 N
*b_snap_/b_spring_*	0.5	0.3	1	3	1.5
*F_cell,e_*	−8 to 1 N	−20 to −11 N	8 to 26 N	31 to 59 N	23 to 50 N
expected behavior	ambiguous	self-recovering	bistable	bistable	bistable

**Table 2:** Characteristic forces *F_1_* to *F_5_* and their standard deviation based on the force–displacement curve of the 4th load cycle. The numbers 1 to 5 refer to the different states during compression and unloading (cf. Figure 3a).

Unit Cell	*F*_1_ [N]	*F*_2_ [N]	*F*_3_ [N]	*F*_4_ [N]	*F*_5_ [N]
V1.2	22.2 ± 0.4	18.5 ± 0.7	28.2 ± 1.5	9.6 ± 0.4	12.2 ± 0.1
V1.3	27.5 ± 0.6	27.2 ± 0.6	37.7 ± 1.2	16.4 ± 0.5	17.9 ± 0.1
V2.2	30.8 ± 1.2	17.6 ± 0.6	27.9 ± 1.3	−1.3 ± 1.6	10.6 ± 1.0
V3.1	33.9 ± 0.6	6.8 ± 0.6	16.4 ± 0.6	−20.1 ± 0.2	5.8 ± 0.7
V3.2	39.4 ± 1.5	17.2 ± 1.3	28.1 ± 1.5	−10.7 ± 0.7	9.6 ± 0.4

**Table 3:** Damping and stiffness properties of the tested unit cells.

Unit Cell	*U* [mJ]	Δ*U* [mJ]	*η* [-]	*E_unit,cell_* [MPa]
V1.2	24.7 ± 1.4	9.4 ± 0.4	0.0622 ± 0.0019	2.1 ± 0.2
V1.3	34.8 ± 0.3	11.0± 0.2	0.0502 ± 0.0008	2.7 ± 0.1
V2.2	31.0 ± 1.8	22.7 ± 2.4	0.116 ± 0.007	2.0 ± 0.1
V3.1	26.0 ± 1.5	31.2 ± 2.0	0.1914 ± 0.0016	1.2 ± 0.02
V3.2	36.0 ± 0.8	33.5 ± 1.0	0.1478 ± 0.0012	2.2 ± 0.1

**Figure A1:**
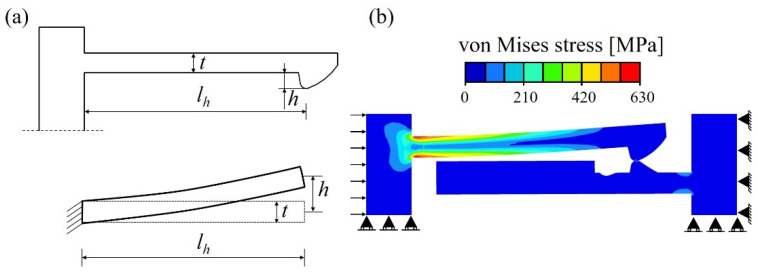
Model and simulations for dimensioning the snap-fit mechanism. (**a**) Contour of a snap-fit hook and simplified cantilever beam, (**b**) von Mises stresses visualized through numerical simulation with implemented boundary conditions.

**Figure A2:**
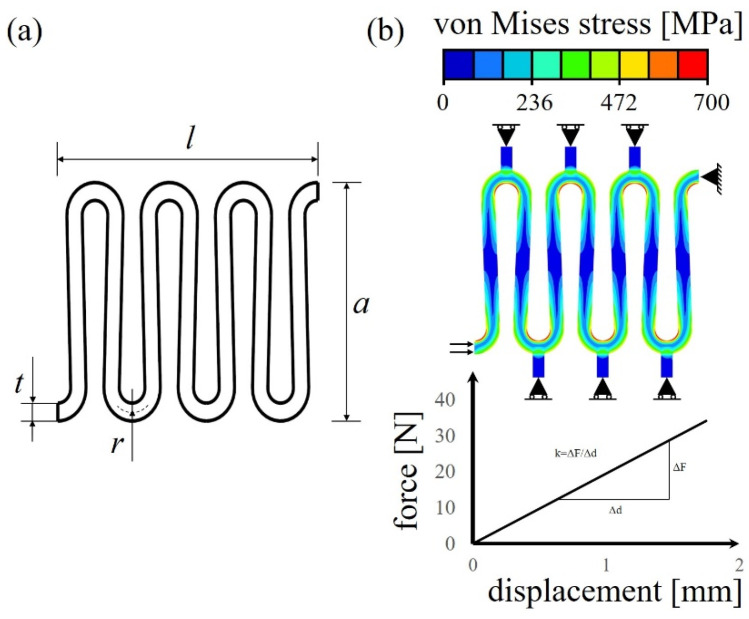
Model and simulations for dimensioning the spring mechanism. (**a**) Contour of a spring design for additive manufacturing, (**b**) von Mises stresses for a spring width of 3 mm at a displacement of 1.75 mm with implemented boundary conditions and force–displacement curve of the compressed spring.

## Data Availability

The data and results involved in this study have been presented in detail in the paper.

## Appendix A

The parameter space available for the unit cell concept cannot be explored without guidance through analytical and numerical simulations. The dimensioning of the unit cell was therefore performed based on initial analytical calculations and detailed numerical simulations to generate a unit cell model able to deform elastically in the range of compression relevant for the experiments.

### Appendix A.1. Dimensioning of Snap-Fit Mechanism

Initial estimates of the dimensioning of the snap-fit mechanism were made using a simplified model of a cantilever beam under an end load (Figure A1a). Considering the goal of miniaturization of the unit cell and the minimum dimensions to be manufactured by the additive manufacturing process, the length *l_h_* and the thickness t of the snap-fit arm were determined empirically. To predict the permissible hook depth *h_max_* of the snap-fit, the maximum elastic deflection of the snap-fit arm is calculated analytically.

(A1) $$h_{max}=\frac{2R_{p0.2}l_{c}{}^{2}}{3Et}$$

*h_max_* can be expressed as a function of the assumed beam length *l_h_* and the modulus of elasticity *E*. In addition, the yield strength *R_p_*_0.2_ serves as a boundary condition for the bending stresses at the maximum centroidal distance *t*/2.

To predict the mechanical behavior of the designed snap-fit mechanism and ensure an elastic behavior of the system, a two-dimensional finite element method was performed to quantify stress distribution in the snap-fits under load and symmetric boundary conditions, as shown in Figure A1b. A change in cross-section width (i.e., snap-fit width) does not influence the maximum stresses when the cantilever is under load and therefore was not considered in the simulation. The maximum stress of 626 MPa did not exceed the material’s yield strength.

### Appendix A.2. Dimensioning of Spring Mechanism

The spring was used for the flexible connection and guidance of the joining partners as well as for storing elastic strain energy. Since spiral springs contain problematic overhangs for additive manufacturing, a spring geometry more suitable for production was designed. The dimensioning includes varying the number and amplitude of the coils as well as the wall thickness, as shown in Figure A2a.

The challenge here is the design of a spring constant which allows the design for a bistable and a self-recovering mechanism when the cell is compressed by a variation of the spring width. The bistability was achieved by a stiffness which leads to a spring force lower than the joining forces of the snap connection that have to be overcome. By relatively increasing the width, the exact opposite effect was triggered, so that the cell returns to its initial state on its own after unloading. At the same time, it is important to dimension the spring with the aim of a purely elastic deformation.

The model of a spring with *N* = 7 windings was discretized with hexahedral elements and, with the help of nonlinear FEM, the loading and unloading of the cell was implicitly calculated by specifying the same displacement as for the snap-fit process in a quasi-static simulation. The geometry parameters shown in Figure A2a are *t* = 0.6 mm, *l* = 10.15 mm, *a* = 8 mm, and *r* = 0.8 mm for the two-dimensional contour to be extruded. Maximum von Mises stresses of 707 MPa result from the simulation with a spring width of 3 mm and applied boundary conditions. A variation of the spring width allows the force level to be adjusted during the elastic deformation without significantly affecting the stresses. By evaluating the force as a function of the displacement, a linear spring characteristic was observed (see Figure A2b). For a selected geometry, the spring constant *k* was determined as a function of the spring width *b_spring_* to predict the spring force dependent on the displacement *d* as follows:

(A2) $$F=\underset{k}{\underbrace{6.5\;\frac{N}{mm^{2}}\times \;b_{spring}}}\times d$$

## Appendix B

During the compression process of the unit cell, the total energy *U_total_*, equivalent to the sum of *U_pot_* and *U_th_*, is absorbed. *U_pot_* is the potential energy resulting from the potential energy of the spring *U_pot,spring_* and the potential energy of the snap-fit mechanism *U_pot,snap-fit_*. *U_th_* represents the thermal energy which is caused by the frictional contact.

The potential energy of the snap-fit mechanism results from the elastic deformation of the frictional elements. Under mechanical load, potential energy *U_pot,snap-fit_* is stored in the form of strain energy in an elastically deformed structure and is defined as

(A3) $$U_{pot,snap-fit}=\int \sigma d\varepsilon$$

The stored potential energy of the spring *U_pot,spring_* is caused by the elastic deformation of the spring element and is described by the linear spring stiffness coefficient k and the displacement *d* caused by the compression.

(A4) $$U_{pot,spring}=\int \sigma d\varepsilon =\frac{1}{2}\;k\;d^{2}$$

In addition to the potential strain energy, the frictional contact leads to dissipation of thermal energy. Assuming that the energy supplied to overcome the Coulombic static and kinetic friction is completely converted into thermal energy, the dissipated thermal energy *U_th_* corresponds to the friction work during the compression progress:

(A5) $$U_{th}≙{\;W}_{fric}=\int \mu_{k}\;F_{n}\left(z\right)\;dz$$

Here, the dependence on the acting normal contact force *F_n_* and the coefficient of friction $\mu_{k}$ for sliding is given. In order to ensure the repeatability of the compression processes of the metamaterial, the structures are designed for purely elastic deformation.

## References

[1] Davoodi M.M., Sapuan S.M., Aidy A., Abu Osman N.A., Oshkour A.A., Wan Abas W. (2012). Development process of new bumper beam for passenger car: A review. Mater. Des..

[2] Caserta G.D., Iannucci L., Galvanetto U. (2011). Shock absorption performance of a motorbike helmet with honeycomb reinforced liner. Compos. Struct..

[3] Di Landro L., Sala G., Olivieri D. (2002). Deformation mechanisms and energy absorption of polystyrene foams for protective helmets. Polym. Test..

[4] Shinagawa M., Shamoto E. (2012). Study on Dynamic Stiffness of Machine Tool with Consideration of Friction Damping in Guide. AMR.

[5] Ashby M.F. (1999). Materials Selection in Mechanical Design.

[6] Gagnon L., Morandini M., Ghiringhelli G.L. (2020). A review of friction damping modeling and testing. Arch. Appl. Mech..

[7] Karayel D., Atali G., Ozkan S. (2018). Design of a New Energy Absorber Based on the Coulomb Friction to Protect Structures Against Impact Loads. Acta Phys. Pol. A.

[8] Wu Z., Xu G., Yang H., Li M. (2021). Analysis of Damping Characteristics of a Hydraulic Shock Absorber. Shock. Vib..

[9] Duym S., Stiens R., Rerybrouck K. (1997). Evaluation of Shock Absorber Models. Veh. Syst. Dyn..

[10] Surjadi J.U., Gao L., Du H., Li X., Xiong X., Fang N.X., Lu Y. (2019). Mechanical Metamaterials and Their Engineering Applications. Adv. Eng. Mater..

[11] Tong X.C. (2018). Functional Metamaterials and Metadevices.

[12] Wu W., Hu W., Qian G., Liao H., Xu X., Berto F. (2019). Mechanical design and multifunctional applications of chiral mechanical metamaterials: A review. Mater. Des..

[13] Kadic M., Milton G.W., van Hecke M., Wegener M. (2019). 3D metamaterials. Nat. Rev. Phys..

[14] Jia Z., Liu F., Jiang X., Wang L. (2020). Engineering lattice metamaterials for extreme property, programmability, and multifunctionality. J. Appl. Phys..

[15] Zadpoor A.A. (2016). Mechanical meta-materials. Mater. Horiz..

[16] Schwerdtfeger J., Wein F., Leugering G., Singer R.F., Körner C., Stingl M., Schury F. (2011). Design of auxetic structures via mathematical optimization. Adv. Mater. Weinheim..

[17] Correa D.M., Seepersad C.C., Haberman M.R. (2015). Mechanical design of negative stiffness honeycomb materials. Integr. Mater..

[18] Dell’Isola F., Seppecher P., Alibert J.J., Lekszycki T., Grygoruk R., Pawlikowski M., Steigmann D., Giorgio I., Andreaus U., Turco E. (2019). Pantographic metamaterials: An example of mathematically driven design and of its technological challenges. Contin. Mech. Thermodyn..

[19] Frenzel T., Kadic M., Wegener M. (2017). Three-dimensional mechanical metamaterials with a twist. Science.

[20] Thompson M.K., Moroni G., Vaneker T., Fadel G., Campbell R.I., Gibson I., Bernard A., Schulz J., Graf P., Ahuja B. (2016). Design for Additive Manufacturing: Trends, opportunities, considerations, and constraints. CIRP Ann..

[21] Fischer S.C.L., Hillen L., Eberl C. (2020). Mechanical Metamaterials on the Way from Laboratory Scale to Industrial Applications: Challenges for Characterization and Scalability. Materials.

[22] Pfaff A., Jäcklein M., Hoschke K., Wickert M. (2018). Designed Materials by Additive Manufacturing—Impact of Exposure Strategies and Parameters on Material Characteristics of AlSi10Mg Processed by Laser Beam Melting. Metals.

[23] Che K., Yuan C., Qi H.J., Meaud J. (2018). Viscoelastic multistable architected materials with temperature-dependent snapping sequence. Soft Matter.

[24] Findeisen C., Hohe J., Kadic M., Gumbsch P. (2017). Characteristics of mechanical metamaterials based on buckling elements. J. Mech. Phys. Solids.

[25] Frenzel T., Findeisen C., Kadic M., Gumbsch P., Wegener M. (2016). Tailored Buckling Microlattices as Reusable Light-Weight Shock Absorbers. Adv. Mater. Weinheim..

[26] Ha C.S., Lakes R.S., Plesha M.E. (2018). Design, fabrication, and analysis of lattice exhibiting energy absorption via snap-through behavior. Mater. Des..

[27] Rafsanjani A., Akbarzadeh A., Pasini D. (2015). Snapping mechanical metamaterials under tension. Adv. Mater..

[28] Shan S., Kang S.H., Raney J.R., Wang P., Fang L., Candido F., Lewis J.A., Bertoldi K. (2015). Multistable Architected Materials for Trapping Elastic Strain Energy. Adv. Mater..

[29] Yang H., Ma L. (2019). Multi-stable mechanical metamaterials by elastic buckling instability. J. Mater. Sci..

[30] Zhang Y., Restrepo D., Velay-Lizancos M., Mankame N.D., Zavattieri P.D. (2019). Energy dissipation in functionally two-dimensional phase transforming cellular materials. Sci. Rep..

[31] Haghpanah B., Shirazi A., Salari-Sharif L., Guell Izard A., Valdevit L. (2017). Elastic architected materials with extreme damping capacity. Extrem. Mech. Lett..

[32] Jiang H., Le Barbenchon L., Bednarcyk B.A., Scarpa F., Chen Y. (2020). Bioinspired multilayered cellular composites with enhanced energy absorption and shape recovery. Addit. Manuf..

[33] Tan X., Chen S., Wang B., Zhu S., Wu L., Sun Y. (2019). Design, fabrication, and characterization of multistable mechanical metamaterials for trapping energy. Extrem. Mech. Lett..

[34] Haghpanah B., Salari-Sharif L., Pourrajab P., Hopkins J., Valdevit L. (2016). Multistable Shape-Reconfigurable Architected Materials. Adv. Mater. Weinheim..

[35] Qiu J., Lang J.H., Slocum A.H. (2004). A Curved-Beam Bistable Mechanism. J. Microelectromech. Syst..

[36] Garland A.P., Adstedt K.M., Casias Z.J., White B.C., Mook W.M., Kaehr B., Jared B.H., Lester B.T., Leathe N.S., Schwaller E. (2020). Coulombic friction in metamaterials to dissipate mechanical energy. Extrem. Mech. Lett..

[37] Salzbrenner B.C., Rodelas J.M., Madison J.D., Jared B.H., Swiler L.P., Shen Y.-L., Boyce B.L. (2017). High-throughput stochastic tensile performance of additively manufactured stainless steel. J. Mater. Process. Technol..

